# Non-glycosylated G protein with CpG ODN provides robust protection against respiratory syncytial virus without inducing eosinophilia

**DOI:** 10.3389/fimmu.2023.1282016

**Published:** 2023-12-19

**Authors:** Eigo Kawahara, Takehiko Shibata, Toshiro Hirai, Yasuo Yoshioka

**Affiliations:** ^1^ Laboratory of Nano-design for Innovative Drug Development, Graduate School of Pharmaceutical Sciences, Osaka University, Osaka, Japan; ^2^ Vaccine Creation Group, Research Institute for Microbial Diseases, Osaka University, Osaka, Japan; ^3^ Department of Microbiology, Tokyo Medical University, Tokyo, Japan; ^4^ Institute for Open and Transdisciplinary Research Initiatives, Osaka University, Osaka, Japan; ^5^ Center for Advanced Modalities and DDS, Osaka University, Osaka, Japan; ^6^ Innovative Vaccine Research and Development Center, The Research Foundation for Microbial Diseases of Osaka University, Osaka, Japan; ^7^ Global Center for Medical Engineering and Informatics, Osaka University, Osaka, Japan; ^8^ Center for Infectious Disease Education and Research, Osaka University, Osaka, Japan

**Keywords:** adjuvant, CpG oligodeoxynucleotide, eosinophil, G protein, respiratory syncytial virus, vaccine

## Abstract

**Introduction:**

Respiratory syncytial virus (RSV) vaccines targeting the fusion glycoprotein (F protein) are highly effective clinically in preventing RSV challenges. The attachment glycoprotein (G protein) is a potentially effective vaccine antigen candidate, as it is important for cell adhesion during infection. However, vaccine-associated enhanced diseases in mice, such as eosinophilic lung inflammation following RSV challenge, are a concern with G protein vaccines. This study aimed to design an effective G protein vaccine with enhanced safety and efficacy by evaluating the efficacy and adverse reactions of vaccines composed of different recombinant G proteins and adjuvants in mice.

**Methods:**

Mice were subcutaneously immunized with glycosylated G protein expressed in mammalian cells (mG), non-glycosylated G protein expressed in *Escherichia coli* (eG), or F protein with or without aluminum salts (alum), CpG oligodeoxynucleotide (CpG ODN), or AddaVax. After vaccination, the levels of G-specific antibody and T-cell responses were measured. The immunized mice were challenged with RSV and examined for the viral load in the lungs and nasal turbinates, lung-infiltrating cells, and lung pathology.

**Results:**

mG with any adjuvant was ineffective at inducing G-specific antibodies and had difficulty achieving both protection against RSV challenge and eosinophilia suppression. In particular, mG+CpG ODN induced G-specific T helper 1 (Th1) cells but only a few G-specific antibodies and did not protect against RSV challenge. However, eG+CpG ODN induced high levels of G-specific antibodies and Th1 cells and protected against RSV challenge without inducing pulmonary inflammation. Moreover, the combination vaccine of eG+F+CpG ODN showed greater protection against upper respiratory tract RSV challenge than using each single antigen vaccine alone.

**Discussion:**

These results indicate that the efficacy of recombinant G protein vaccines can be enhanced without inducing adverse reactions by using appropriate antigens and adjuvants, and their efficacy is further enhanced in the combination vaccine with F protein. These data provide valuable information for the clinical application of G protein vaccines.

## Introduction

The respiratory syncytial virus (RSV) infects almost all infants by the age of 2 years, with repeated reinfections thereafter (1, 2). It infects 64 million people annually worldwide and causes a high rate of lower respiratory tract illness, particularly in infants within the first six months of life and in older adults with underlying medical conditions (3). Recently, severe acute respiratory syndrome coronavirus 2 (SARS-CoV-2) has co-infected with RSV more frequently than with influenza virus or adenovirus, causing severe illness and mortality (4). With the advent of vaccine development in the 1960s, vaccine-associated enhanced disease (VAED) of the formalin-inactivated RSV (FI-RSV) vaccine has been reported clinically in children, wherein approximately 80% of the infected recipients had been hospitalized for severe lower respiratory tract infection, with two fatalities (5). The mechanism of VAED caused by the FI-RSV vaccine is unclear, however, eosinophilia, which is excessive infiltration of eosinophils, has been observed in the lungs of deceased patients (6–8). In addition, mouse models have suggested that eosinophilia due to FI-RSV vaccine-induced T helper 2 (Th2) responses is associated with VAED (9). Thus, for safety, the development of subunit vaccines rather than whole-virus vaccines has attracted attention.

The fusion glycoprotein (F protein) and attachment glycoprotein (G protein) expressed on the RSV surface are considered effective vaccine targets for developing RSV vaccines. In particular, vaccines targeting the F protein, which is involved in the membrane fusion between RSV and host cells (1, 2), have been actively developed (10). The F protein changes from a metastable pre-fusion (pre-F) to a stable post-fusion (post-F) conformation during the fusion process (1, 11). The F protein vaccines in clinical trials have used pre-F as the vaccine antigen, which can induce higher levels of neutralizing antibodies than those by the post-F antigen (10, 12). Two recently approved RSV vaccines have also used recombinant pre-F as the vaccine antigen (13, 14). The pre-F vaccine exhibits an efficacy of > 90% in older adults and approximately 70% within 180 days of birth in infants whose mothers were vaccinated during pregnancy against RSV-related severe lower respiratory tract disease in clinical trials. However, the vaccine efficacy against RSV-related acute respiratory infections is only approximately 60% in older adults and less than 40% within 180 days of birth in infants (14, 15). Therefore, further improvements in vaccines are required.

The G protein, composed of oligomers on the RSV surface, is involved in the adhesion of RSV to host cells, and vaccines targeting the G protein can block the initiation of infection (1, 16–18). The central conserved domain (CCD), which is relatively common in all RSV subtypes, is a neutralizing G protein epitope important for infection (16, 19–21). The CX3C motif within CCD binds to the chemokine receptor CX3CR1 expressed on host airway epithelial cells and is important for adhesion to these cells (22, 23). However, safety is a concern in the development of G protein vaccines. Recombinant G protein expressed in mammalian cells (mG) is excessively modified with O-linked glycans (1). Vaccination with mG, even without the addition of adjuvant, enhances the Th2-type immune response and induces eosinophilia in the lungs after RSV challenge in mice, similar to that observed with FI-RSV vaccines (17, 24, 25). Recently, vaccination with recombinant G protein expressed in *Escherichia coli* (*E.coli*) (eG), with no glycan modifications, along with an adjuvant, has exhibited protection against RSV challenge with lesser induction of Th2 responses and eosinophilia than those with glycosylated mG in mice and cotton rats (17, 26). Therefore, the use of eG as an antigen may be a potential approach to achieve vaccine effects without adverse reactions.

In addition, adjuvants are usually used to enhance immunogenicity and control the Th1/Th2 balance (27). Adjuvants such as aluminum salts (alum), oligodeoxynucleotide (ODN) with unmethylated cytosine-phosphate-guanine (CpG ODN), which is a toll-like receptor (TLR) 9 ligand (28), and squalene oil-in-water emulsions are used in licensed vaccines (29–31). Because the Th2 response is considered a cause of VAED in recombinant G protein vaccines, controlling the Th1/Th2 balance using these adjuvants in combination is a possible solution.

This study aimed to explore G protein vaccines with enhanced efficacy and safety, by comparing the efficacy and adverse reactions of mG and eG with those of different common adjuvants.

## Materials and methods

### Mice

Female BALB/c mice (6–7-week-old) were purchased from SLC (Hamamatsu, Japan). The mice were maintained in a room with a 12 h/12 h light/dark cycle (lights on, 8:00 a.m.; lights off, 8:00 p.m.) and provided *ad libitum* access to food and water. All animal experiments were conducted according to the guidelines of Osaka University for the ethical treatment of animals and were subject to approval by the Animal Care and Use Committee of the Research Institute for Microbial Diseases, Osaka University, Japan (protocol numbers: BIKEN-AP-R01-15-2, BIKEN-AP-R02-14-5).

### Antigen expression and purification

Sequences for the G and F proteins were derived from RSV (strain A2) (GenBank accession number: AAB59857.1, G protein; GenBank accession number: AAB59858.1, F protein). Plasmid vectors encoding the human codon-optimized sequence of the G protein ectodomain (amino acids 67–298) with an N-terminal hexahistidine tag (His-tag) or F protein were provided by Dr. Tadaki Suzuki (Department of Pathology, National Institute of Infectious Diseases, Tokyo, Japan). The F protein is based on DS-Cav1 and consists of amino acids 1–513 mutated at P102A, S155C, S190F, V207L, S290C, I379V, and M447V, with a C-terminal foldon (GYIPEAPRDGQAYVRKDGEWVLLSTFL) of fibritin from bacteriophage T4 and a His-tag (11, 32). Recombinant mG and F proteins were generated using the Expi293 Expression System (Thermo Fisher Scientific, Hampton, NH, USA) according to the manufacturer’s instructions. Briefly, Expi293F cells cultured in Expi293 Expression Medium in 125 mL Erlenmeyer flasks were diluted to 3.0 × 10^6^ cells/mL. Then, 30 μg of plasmid vector was mixed with ExpiFectamine 293 Reagent and transfected into 7.5 × 10^7^ Expi293F cells in 25 mL medium. After incubation at 37°C, 8% CO_2_ on a reciprocating shaker (120 rpm) for 20 h, ExpiFectamine 293 Transfection Enhancer 1 and 2 were added. Six days later, the supernatant was collected via centrifugation at 8,000 × *g* for 10 min. The *E.coli* codon-optimized sequence of G protein ectodomain was cloned into the pCold3 vector (Takara Bio, Inc., Kusatsu, Shiga, Japan). The recombinant eG protein was produced by adding 0.2 mM isopropyl-β-D-1-thiogalactopyranoside to transformed BL21 (DE3) with shaking for 24 h at 15 °C. After incubation, the *E.coli* cells were collected via centrifugation at 8,000 × *g* for 10 min and resuspended in 100 mM NaCl, 5 mM imidazole, and 20 mM Tris-HCl containing Protease Inhibitor Cocktail (Nacalai Tesque, Kyoto, Japan). The cells were lysed using sonication, and the supernatant was collected via centrifugation at 8,000 × *g* for 60 min. All soluble recombinant mG, eG, and F proteins were purified using an AKTA explorer chromatography system (GE Healthcare, Diegem, Belgium) with a Ni-Sepharose HisTrap FF column (GE Healthcare) and a Superose 6 Increase 10/300 GL column (GE Healthcare). For eG, endotoxins were removed using an EndoTrap HD 5/1 (LIONEX, Braunschweig, Germany). The endotoxin level in eG (<0.05 endotoxin unit [EU]) was checked using the Limulus Color KY Test (FUJIFILM Wako Pure Chemical Corporation, Osaka, Japan). Protein purity was confirmed using sodium dodecyl sulfate polyacrylamide gel electrophoresis (SDS-PAGE) and Coomassie Brilliant Blue staining.

### RSV progression and purification

HEp-2 cells and the RSV (strain A2) were kindly provided by Dr. Takehiko Shibata (Department of Microbiology, Tokyo Medical University, Tokyo, Japan). HEp-2 cells were cultured in Dulbecco’s modified Eagle’s medium (high glucose) supplemented with 10% heat-inactivated fetal bovine serum (FBS), 1% penicillin, and 1% streptomycin. The cells were maintained at 37°C in a humidified incubator with 5% CO_2_. RSV was propagated by infecting sub-confluent HEp-2 monolayers. The viruses were collected 5 days post-infection by freeze-thawing cells and centrifuged twice at 700 × *g* for 5 min at 4°C to collect the supernatant. Harvested viruses were centrifuged at 71,000 × *g* for 3.5 h at 4°C; the pellet was resuspended in phosphate buffered saline (PBS), and stored at −80°C for a maximum of 1 year. Stocked virus titers were determined using a plaque assay in HEp-2 cells. Briefly, diluted solutions containing the viruses were added to confluent HEp-2 cells in 24-well plates. After incubation at 37°C with 5% CO_2_ for 2 h, the supernatant was aspirated and the culture medium supplemented with 0.6% carboxymethyl cellulose was added to the wells. After incubation at 37°C with 5% CO_2_ for 3 days, the supernatant was aspirated, and the cells and viruses were fixed with methanol at −80°C. The plates were incubated in PBS containing 5% skim milk (Becton Dickinson, Franklin Lakes, New Jersey, USA) at 37°C for 1 h. Then, the plates were incubated with anti-RSV polyclonal antibody (catalog numbers: AB1128, dilution 1/500; Merck Millipore, Darmstadt, Germany) at 37°C for 1 h, followed by incubation with horseradish peroxidase (HRP)–conjugated donkey anti-sheep IgG polyclonal antibody (catalog numbers: STAR88P, dilution 1/250; Bio-rad, Hercules, CA, USA) at 37°C for 1 h. After incubation, a color reaction was developed using PBS containing 0.3 mg/mL 4-chloro-1-napthol (Tokyo Chemical Industry Co., Ltd., Tokyo, Japan) and 0.03% hydrogen peroxide. Plaques were counted visually. All experiments involving viruses were subject to approval by the Institutional Review Board of the Research Institute for Microbial Diseases, Osaka University (protocol number: BIKEN-00224-002).

### Vaccine and RSV challenge

BALB/c mice were subcutaneously immunized with mG, eG, or F protein (1 μg/mouse) with or without Alhydrogel adjuvant 2% (alum; InvivoGen, San Diego, CA, USA) (50 μg/mouse), CpG ODN K3 (5′-atcgactctcgagcgttctc-3′) (GeneDesign, Osaka, Japan) (10 μg/mouse), or AddaVax (InvivoGen) (25 μL/mouse) at the base of the tail on days 0 and 21. On day 28, plasma and spleen were collected. On day 31, the mice were challenged intranasally with 1.0 × 10^5^ plaque-forming units (PFU) of RSV in 30 μL of PBS (15 μL to each nostril) under anesthesia. Five days after the RSV challenge, the mice were anesthetized and euthanized. The right lungs were weighed. The right lung and nasal turbinate were collected using TRIzol Reagent (Thermo Fisher Scientific) followed by tissue homogenization for RNA extraction. The left lung was collected in Roswell Park Memorial Institute (RPMI)1640 medium containing 5% FBS and 20 mM HEPES and analyzed for infiltrating cells using flow cytometry.

### Enzyme-linked immunosorbent assay

Antigen-specific immunoglobulin (Ig)G, IgG1, IgG2a, and IgG2b in plasma was detected using an enzyme-linked immunosorbent assay (ELISA). Microplates (96-well half area; Corning, NY, USA) were coated with mG or F in carbonate buffer (1 μg/mL) for 12–18 h at 4°C. The coated plates were incubated with 1% Block Ace (DS Pharma Biomedical, Osaka, Japan). The plasma was diluted with 0.4% Block Ace and added to antigen-coated wells. After incubation for 2 h at 20–25°C, the plates were incubated in a solution containing HRP–conjugated goat anti-mouse IgG (catalog number: AP503, dilution 1/5,000; Merck Millipore), IgG1 (catalog numbers:1070-05, dilution 1/8,000; SouthernBiotech, Birmingham, USA), IgG2a (catalog number: ab97245, dilution 1/5,000; Abcam, Cambridge, UK), and IgG2b (catalog numbers:1090-05, dilution 1/5,000; SouthernBiotech) for 1 h at 20–25°C. After incubation, color was developed with tetramethylbenzidine (Nacalai Tesque) and arrested using 2 N H_2_SO_4_; the difference in optical density (OD) between 450 nm and 570 nm (OD_450-570_) was measured using a microplate reader (Power Wave HT, BioTek, Winooski, VT, USA). The endpoint titer was the maximum dilution rate at which the OD value minus the background value was greater than or equal to 0.1.

### T cell re-stimulation

Seven days after the second immunization, the spleen was mashed and filtered through a 70 μm cell strainer to obtain a single cell suspension, which was hemolyzed with ACT lysis buffer containing 8.3 g/L NH_4_Cl and 0.01 M Tris-HCl at pH 7.5. Splenocytes (1–3 × 10^6^ cells) were re-stimulated with 10 μg/mL mG for 3 days at 37°C in 96-well U-bottom plates. A protein transport inhibitor cocktail (Thermo Fisher Scientific) was added to the cells and incubated for 5 h. For surface antigen staining, the cells were then incubated with anti-mouse CD16/CD32 antibody (clone: 93, catalog numbers: 101302, dilution 1/200; BioLegend, San Diego, CA, USA), Fixable Viability Dye eFluor 780 (catalog numbers: 65-0865-18, dilution 1/1000; Thermo Fisher Scientific), APC anti-mouse CD3ϵ antibody (clone: 145-2C11, catalog numbers: 100312, dilution 1/200; BioLegend), Alexa Fluor 700 anti-mouse CD4 antibody (clone: GK1.5, catalog numbers: 100430, dilution 1/500; BioLegend), Brilliant Violet 510 anti-mouse/human CD44 antibody (clone: IM7, catalog numbers: 103044, dilution 1/200; BioLegend), and Brilliant Violet 605 anti-mouse CD8a antibody (clone: 53-6.7, catalog numbers: 100744, dilution 1/200; BioLegend) in PBS containing 2% FBS, 1 mM EDTA, and 0.05% sodium azide for 15 min at 4°C in dark. For intracellular cytokine staining, the cells were subsequently treated with BD Cytofix/CytopermTM Fixation/Permeabilization solution Kit (BD Biosciences, Sparks, MO, USA) in accordance with the manufacturer’s instructions, followed by incubation with Brilliant Violet 421 anti-mouse interleukin (IL)-4 antibody (clone: 11B11, catalog numbers: 504120, dilution 1/200; BioLegend), Brilliant Violet 605 anti-mouse interferon (IFN)-γ antibody (clone: XMG1.2, catalog numbers: 505840, dilution 1/200; BioLegend), PE anti-mouse/human IL-5 antibody (clone: TRFK5, catalog numbers: 504304, dilution 1/200; BioLegend), and PE-cyanine7 anti-mouse/human IL-13 antibody (clone: eBio13A, catalog numbers: 25-7133-82, dilution 1/200; Thermo Fisher Scientific) for 30 min at 4°C in dark. Data were acquired using an Attune NxT Flow Cytometer (Thermo Fisher Scientific) and analyzed using the FlowJo software version 10.9 (FlowJo LLC, Ashland, Oregon, USA).

### RNA extraction and real-time reverse transcription polymerase chain reaction

Right lungs were excised from mice 5 days after RSV challenge and homogenized in 1 mL TRIzol. RNA was purified using chloroform and isopropanol and the RNA pellets were washed with 70% ethanol, dried for 10 min, and dissolved in nuclease-free water. cDNA was synthesized using the ReverTra Ace qPCR RT Master Mix (Toyobo, Osaka, Japan). Real-time reverse transcription polymerase chain reaction (RT-PCR) was performed by amplifying the target genes and *Gapdh* mRNA as a reference gene using a Light Cycler 480-II (Roche Diagnostics, Tokyo, Japan) with primers (**Supplementary Table 1**) and LightCycler 480 SYBR Green I Master (Roche Diagnostics). Absolute quantification of RSV levels was performed using a plasmid copy number standard for the RSV nucleoprotein gene.

### Analysis of infiltrating cells into the lungs

Left lungs excised from mice 5 days post infection, sliced, and incubated in RPMI1640 medium containing 5% FBS, 20 mM HEPES, 200 U/mL collagenase IV (Thermo Fisher Scientific), and 100 U/mL DNase I (FUJIFILM Wako Pure Chemical Corporation) for 60 min at 37°C with shaking. After incubation, the lungs were dissociated using a gentleMACS Dissociator (Miltenyi Biotech, Bergisch Gladbach, Germany), according to the manufacturer’s protocol. Single-cell suspensions were prepared by gently pushing the tissue through a 70-μm cell strainer and hemolyzing with ACT lysis buffer. For surface antigen staining, cells isolated from the lungs were incubated with anti-mouse CD16/CD32 antibody, Fixable Viability Dye eFluor 780, FITC anti-mouse Ly-6G antibody (clone: 1A8, catalog numbers: 127606, dilution 1/500; BioLegend), APC anti-mouse Siglec-F antibody (clone: REA798, catalog numbers: 130-112-175, dilution 1/200; Miltenyi Biotech), Alexa Fluor 700 anti-mouse CD4 antibody, Brilliant Violet 421 anti-mouse I-A/I-E antibody (clone: M5/114.15.2, catalog numbers: 107632, dilution 1/200; BioLegend), Brilliant Violet 510 anti-mouse/human CD11b antibody (clone: M1/70, catalog numbers: 101263, dilution 1/200; BioLegend), Brilliant Violet 605 anti-mouse CD8a antibody, PE anti-mouse CD45 antibody (clone: 30-F11, catalog numbers: 103106, dilution 1/200; BioLegend), and PE/Cyanine7 anti-mouse CD3 antibody (clone: 17A2, catalog numbers: 100220, dilution 1/200; BioLegend) in PBS containing 2% FBS, 1 mM EDTA, and 0.05% sodium azide for 15 min at 4°C in dark. Data were acquired on an Attune NxT Flow Cytometer and analyzed using the FlowJo software version 10.9.

### Serum adoptive transfer

Seven days after the second immunization, serum was collected from the vaccine-immunized or PBS-treated mice. A pooled serum sample was heat-inactivated and mixed with 1.0 × 10^5^ PFU of RSV *in vitro*, and this mixture was intranasally administered to naive mice in 30 μL PBS under anesthesia. Five days later, mice were anesthetized and euthanized. The right lung was collected using TRIzol reagent, followed by tissue homogenization for RNA extraction.

### Statistical analyses

Statistical analysis was performed using the GraphPad Prism 9 (GraphPad Software, San Diego, CA, USA). Data are presented as means ± standard deviation (SD) or as medians. Significant differences were determined using Tukey’s test or Dunnett’s test after One-way ANOVA. Statistical significance was set at *P* < 0.05.

## Results

### eG+CpG ODN or AddaVax induced high levels of G-specific IgG

**Figure 1:**
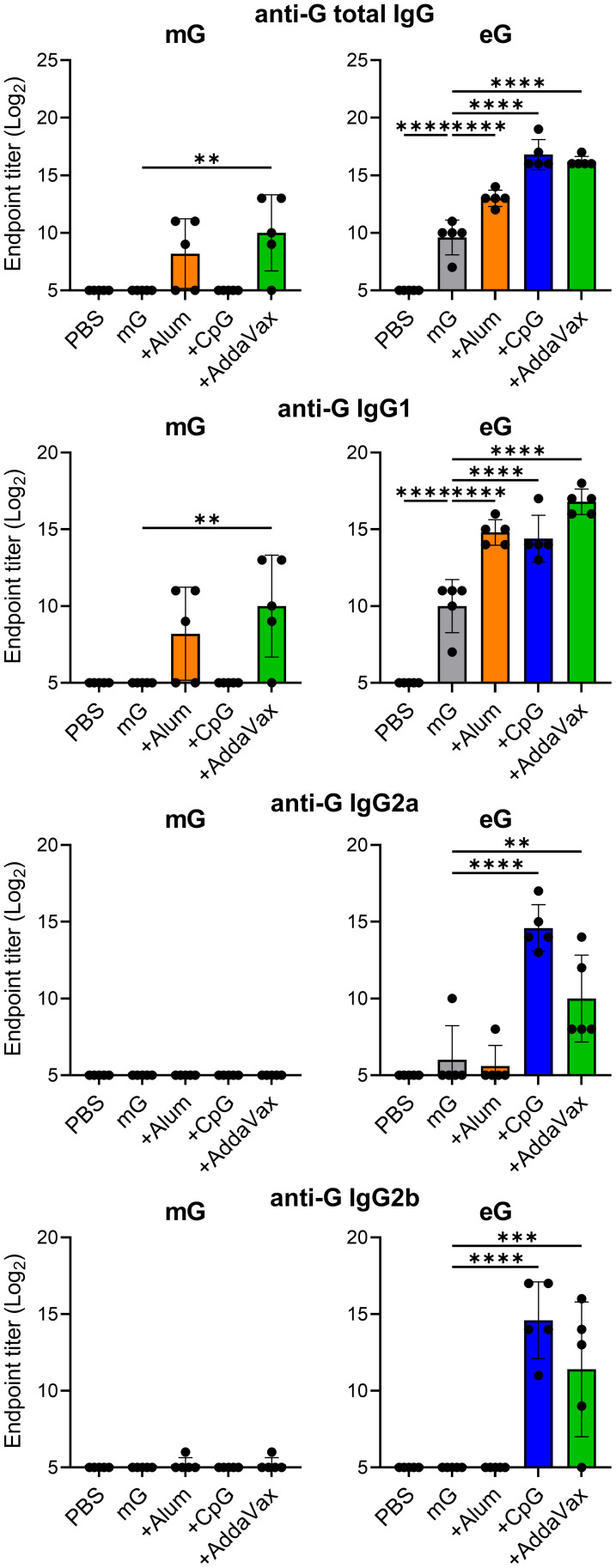
Antibody responses to recombinant G protein expressed in mammalian cells (mG) or recombinant G protein expressed in *E. coli* (eG) vaccines. Mice were immunized subcutaneously with 1 µg mG or eG alone, +50 µg alum, 10 µg cytosine-phosphate-guanine oligodeoxynucleotide (CpG ODN), or 50% AddaVax, or phosphate buffered saline (PBS) as a control on days 0 and 21. Plasma was collected 7 days after the second immunization. The endpoint titers of G-specific total IgG, IgG1, IgG2a, and IgG2b in plasma were evaluated using enzyme linked immunosorbent assay (ELISA) 7 days after the second immunization with mG or eG vaccines. Each experiment was performed twice. n = 5 per group. Data are presented as the means ± standard deviation (SD). ^**^ *P* < 0.01, ^***^ *P* < 0.001, ^****^ *P* < 0.0001 as indicated using Dunnett’s test compared with mG alone or eG alone.

To verify the immunogenicity of G proteins with or without their glycans, we prepared an extracellular domain of the G protein expressed in mammalian cells or *E.coli*. The extracellular domain of the G protein was estimated from the amino acid sequence to be approximately 30 kDa; however, it is approximately 90 kDa owing to the addition of a large amount of glycans when it is produced using mammalian cells (33). A previous report indicated that the extracellular domain of G protein forms oligomers using mammalian cells or *E. coli* (17). Consistent with these findings, our size exclusion gel filtration chromatography revealed that both mG and eG form oligomers (**Supplementary Figure 1A**). SDS-PAGE identified eG monomer at approximately 30 kDa and mG monomer at approximately 90 kDa, indicating that mG contained a large number of glycans (**Supplementary Figure 1B**). The results of size exclusion gel filtration chromatography and SDS-PAGE confirmed that mG and eG were purified free of impurities. Specifically, for eG, endotoxin was also removed using an endotoxin removal column. Vaccine efficacy was compared using different common adjuvants, such as alum, CpG ODN, and AddaVax. As alum is typically used in vaccines at 10 to 50 times the amount of antigen, we used 50 times the amount of alum relative to the amount of antigen. CpG ODN induces splenomegaly in a dose-dependent manner (34). We used 10 μg of CpG ODN because we observed that while mG or eG plus 50 μg of CpG ODN induced splenomegaly, mG or eG plus 10 μg of CpG ODN did not (data not shown). AddaVax is a squalene-based oil-in-water nanoemulsion similar to MF59 (27, 35). Because MF59 is typically used in vaccines at a concentration of 50–100% (vol:vol), we used 50% (vol:vol) of AddaVax. Mice were vaccinated with 1 µg mG or eG alone or with each adjuvant subcutaneously twice, on day 0 and day 21. The plasma levels of G-specific total IgG, IgG1, IgG2a, and IgG2b in vaccinated mice on day 28 were compared using ELISA (**Figure 1**). Because G protein on the RSV surface is modified with glycans such as mG, we used mG to coat the antigen in ELISA, for detecting G-specific antibodies. Neither mG alone nor mG+CpG ODN nor mG+alum induced detectable G-specific total IgG, IgG1, IgG2a, and IgG2b responses. However, compared to mG alone, mG+AddaVax significantly increased G-specific total IgG and IgG1 levels. In contrast, compared to the PBS-treated group, eG alone substantially increased G-specific total IgG and IgG1 levels. CpG ODN and AddaVax increased the immunogenicity of eG in terms of G-specific total IgG and IgG1 as well as IgG2a and IgG2b, whereas alum increased only G-specific total IgG and IgG1 levels relative to eG alone. These results indicated that compared to mG vaccines, eG vaccines exhibited a superior ability to induce antibodies, particularly eG+CpG ODN or AddaVax, which induced high levels of G-specific IgGs.

### mG and eG+CpG ODN did not induce G-specific Th2 cells but induced G-specific IFN-γ-producing CD4^+^ T cells and CD8^+^ T cells

**Figure 2:**
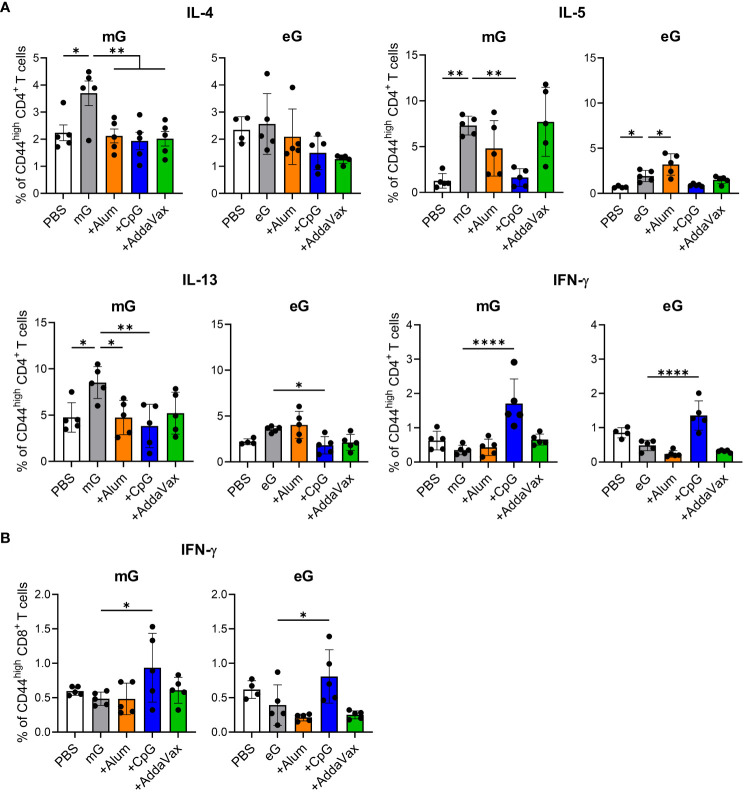
T cell responses to mG or eG vaccines. **(A, B)** Splenocytes from mice immunized with mG or eG vaccines were incubated in the presence of mG for 3 days and with a transport inhibitor for the final 5 h of culture. The intracellular cytokine levels in **(A)** CD44^high^ CD4^+^ T cells and **(B)** CD44^high^ CD8^+^ T cells were evaluated using flow cytometry. **(A, B)** Each experiment was performed twice. n = 4–5 per group. Data are presented as the mean ± SD. ^*^ *P* < 0.05, ^**^ *P* < 0.01, ^****^ *P* < 0.0001 as indicated using Dunnett’s test compared with mG alone or eG alone. IL, interleukin; IFN, interferon.

To evaluate T cell responses induced by the mG and eG vaccines, post-vaccination splenocytes were re-stimulated with mG, because G protein on the RSV surface is modified with glycans such as mG. We then analyzed G-specific CD4^+^ T cells as Th2 cytokines (i.e., IL-4, IL-5, and IL-13) or Th1 cytokine (i.e., IFN-γ)-producing cells using flow cytometry (**Figure 2A**, **Supplementary Figure 2**). Compared to the PBS-treated group, mG alone significantly induced CD4^+^ T cells to produce Th2 cytokines, but not Th1 cytokines, whereas eG alone significantly induced only IL-5-inducing CD4^+^ T cells (**Figure 2A**). Compared to the antigen alone in both mG and eG vaccines, the addition of CpG ODN induced IFN-γ-producing CD4^+^ T cells, but not Th2 cytokine-producing CD4^+^ T cells (**Figure 2A**). The addition of alum did not enhance Th1 or Th2 cytokine-producing CD4^+^ T cells in the mG vaccine but significantly enhanced IL-5-producing CD4^+^ T cells in the eG vaccine (**Figure 2A**). AddaVax did not enhance Th1 or Th2 cytokine-producing CD4^+^ T cells in either the mG or eG vaccines (**Figure 2A**). We also analyzed IFN-γ-producing CD8^+^ T cells (**Figure 2B**, **Supplementary Figure 2**). Compared to the PBS-treated group, neither mG alone nor eG alone induced IFN-γ-producing CD8^+^ T cells (**Figure 2B**). Compared to the antigen alone in both mG and eG vaccines, the addition of CpG ODN significantly induced IFN-γ-producing CD8^+^ T cells, but the addition of alum or AddaVax did not induce IFN-γ-producing CD8^+^ T cells (**Figure 2B**). These results suggest that T cell responses of the mG vaccines involved inducing more of Th2 cytokines than those by the eG vaccines. In addition, although neither mG nor eG alone induced IFN-γ-producing CD4^+^ T cells and CD8^+^ T cells, the addition of CpG ODN induced IFN-γ-producing CD4^+^ T cells and CD8^+^ T cells.

### mG+AddaVax or eG+any adjuvant remarkably protected against RSV challenge

**Figure 3:**
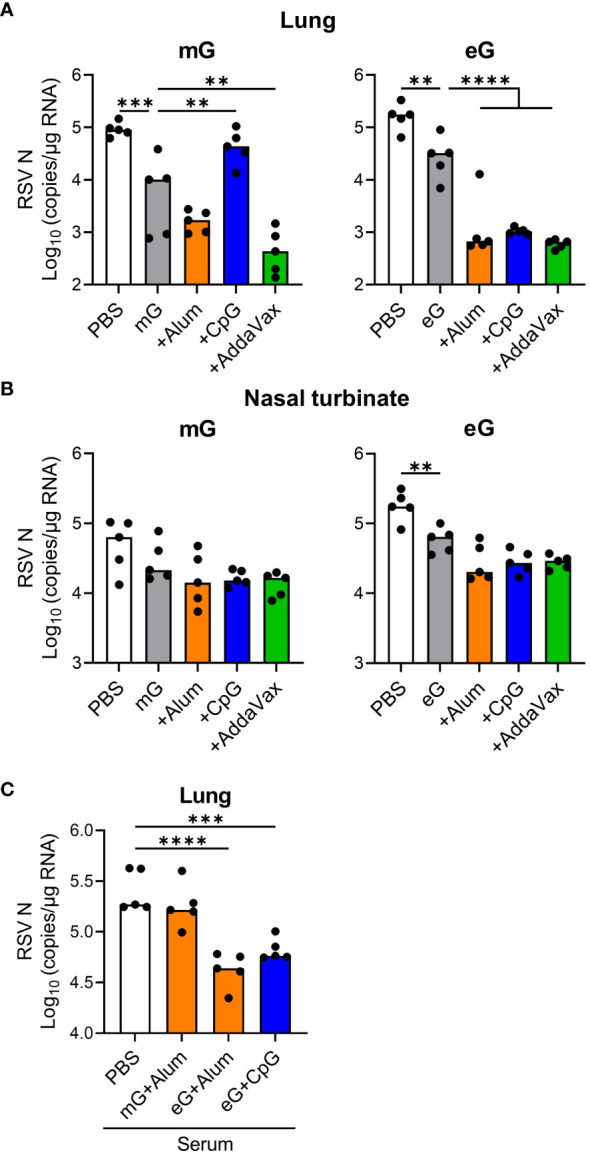
Protective effect of eG or mG vaccines against RSV challenge. **(A, B)** Mice immunized with mG or eG vaccines were challenged intranasally with 1.0 × 10^5^ plaque-forming units (PFU) of RSV 10 days after the second immunization. At 5 days post infection, **(A)** the lungs and **(B)** nasal turbinates were collected, and viral loads were determined using real-time reverse transcription polymerase chain reaction (RT-PCR) from the mRNA levels of RSV nucleoprotein. **(C)** Mixture of 1.0 × 10^5^ PFU RSV and 4-fold diluted serum from PBS-treated control mice or immunized mice was administered intranasally into naive mice. At 5 days post infection, right lungs were collected, and viral loads were determined using real-time RT-PCR for the mRNA levels of RSV nucleoprotein. **(A–C)** Each experiment was performed twice. n = 5 per group. Data are presented as the median. ^**^ *P* < 0.01, ^***^ *P* < 0.001, ^****^ *P* < 0.0001 as indicated using Dunnett’s test compared with **(A, B)** mG alone, eG alone, or **(C)** PBS.

To evaluate the protective effects of mG and eG vaccines against RSV, we infected vaccinated mice intranasally with RSV and measured viral load in the lungs and nasal turbinates. Because of the low sensitivity of the plaque assay to detect virus, we were unable to detect virus in the lungs and nasal sinuses of most vaccinated groups after RSV challenge and could not compare the efficacy of each vaccine. Therefore, we used real-time RT-PCR to quantify the viral load in the tissues. We confirmed that the lung viral load quantified using real-time RT-PCR correlates with the lung viral load quantified using plaque assay when naive mice are infected with RSV (**Supplementary Figures 3A, B**). As naive mice exhibited maximum viral load in the lungs and nasal turbinates on day 5 after RSV infection (**Supplementary Figures 3A–C**), the protective effects of the vaccines were evaluated on day 5 after RSV challenge. In the lungs, the viral load was significantly reduced by mG and eG alone compared to that in the PBS-treated group (**Figure 3A**). The addition of CpG ODN or alum did not reduce the viral load compared with the mG vaccine antigen alone, but significantly reduced the viral load using the eG vaccine (**Figure 3A**). AddaVax significantly reduced the viral load using, both, mG and eG vaccines (**Figure 3A**). In the nasal turbinates, compared with the PBS-treated group, mG alone did not protect against RSV challenge, whereas eG alone did (**Figure 3B**). mG or eG+any adjuvant did not reduce the viral load relative to that with the antigen alone (**Figure 3B**). Further, to determine the role of antibodies in vaccine-induced protection against RSV challenge, serum from the mice immunized with vaccines (i.e., mG+alum, eG+alum, or CpG ODN) that protected against the RSV challenge, or serum from PBS-treated mice as control, was mixed with RSV and administered intranasally to naive mice. The viral load in the lungs was significantly reduced on using serum from mice vaccinated with eG+alum or CpG ODN, compared to serum from PBS-treated mice. However, serum from mice vaccinated with mG+alum did not reduce the viral load (**Figure 3C**), in correlation with their levels of G-specific antibodies (**Figure 1**). Thus, mG+AddaVax, or eG+any adjuvant, could remarkably protect against RSV challenge. In particular, remarkable protection against RSV challenge was demonstrated by eG vaccines, compared to that by mG vaccines.

### eG+CpG ODN protected mice against RSV challenge without pulmonary inflammation

**Figure 4:**
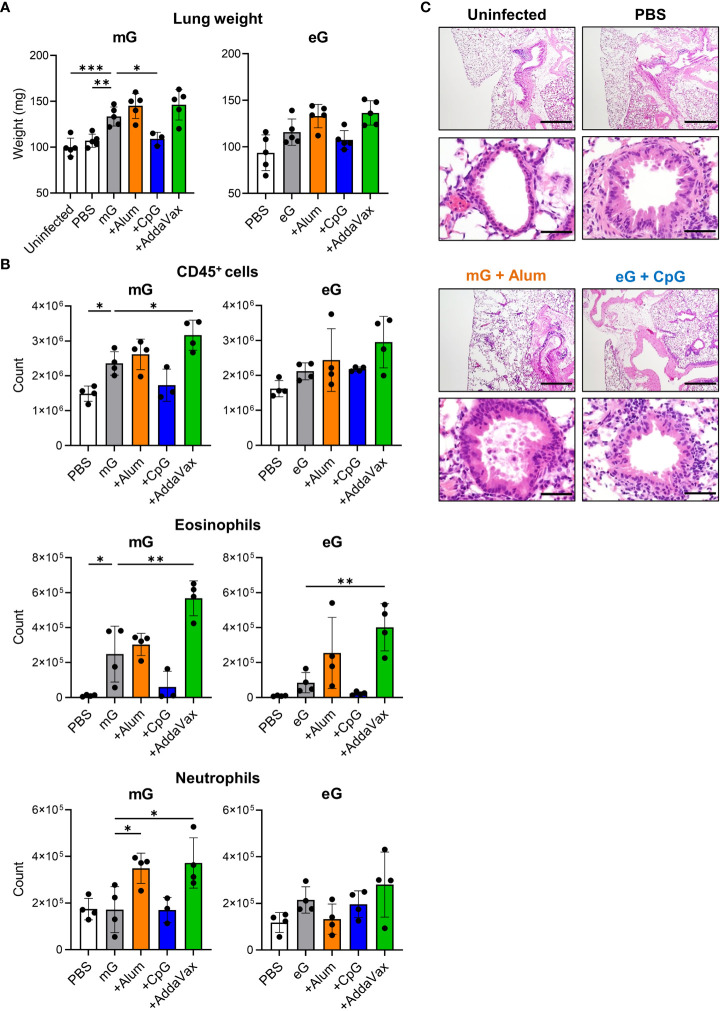
Evaluation of lung inflammation after RSV challenge following mG or eG vaccines. **(A-C)** Mice immunized with mG or eG vaccines were challenged intranasally with 1.0 × 10^5^ PFU of RSV 10 days after the second immunization. At 5 days post infection, lungs were collected. **(A)** Right lung weight. **(B)** Flow cytometry analysis of infiltrating cells in the left lungs. **(C)** Images of hematoxylin-and-eosin-stained lung sections. Scale bars, 200 μm (upper), 50 μm (lower). **(A-C)** Each experiment was performed twice. **(A)** n = 3–5 per group. **(B)** n = 3–4 per group. **(A, B)** Data are presented as the mean ± SD. ^*^ *P* < 0.05, ^**^ *P* < 0.01, ^***^ *P* < 0.001 as indicated using Dunnett’s test compared with mG alone or eG alone.

Further, we examined lung pathology and infiltrating cells after RSV challenge in mice immunized with the mG and eG vaccines (**Supplementary Figure 4A**). Compared to the uninfected and PBS-treated groups, mG alone increased the lung weight and levels of lung-infiltrating CD45^+^ cells and eosinophils, but eG alone did not (**Figures 4A, B**). The lung weight and levels of lung-infiltrating CD45^+^ cells, eosinophils, and neutrophils did not increase on the addition of CpG ODN, compared with the antigen alone in either mG or eG vaccines, but significantly reduced lung weight in the mG vaccine (**Figures 4A, B**). The addition of alum or AddaVax did not increase the lung weight using either the mG or eG vaccines (**Figure 4A**). For lung-infiltrating cells, the addition of alum significantly increased only neutrophil levels in the mG vaccine, but not in the eG vaccine (**Figure 4B**). The addition of AddaVax significantly increased the levels of CD45^+^ cells, eosinophils, and neutrophils in the mG vaccine, but only eosinophil levels increased in the eG vaccine (**Figure 4B**). T cell infiltrations in the lungs were similar in all the vaccinated mice (**Supplementary Figure 4B**). In addition, we compared lung pathology after RSV challenge. Because mG+alum increased lung weight and levels of lung-infiltrating cells after RSV challenge, we used the mG+alum-vaccinated group as a positive control with higher lung inflammation to compare lung pathology with that of eG+CpG ODN, which protects against RSV challenge without inducing lung-infiltrating cells. The lung pathology of mG+alum-vaccinated mice after RSV challenge showed more cellular infiltration and fibrosis than that in uninfected and PBS-treated mice (**Figure 4C**). However, the degree of lung pathology in eG+CpG ODN-vaccinated mice was milder than that in mG+alum-vaccinated mice and similar to that in PBS-treated mice (**Figure 4C**). In summary, eG vaccines tended to induce lower levels of lung-infiltrating CD45^+^ cells and eosinophils following RSV challenge than that by mG vaccines. In addition, eG+CpG ODN protected against RSV challenge without pulmonary inflammation, whereas mG+Alum, AddaVax, or eG+AddaVax protected against RSV challenge but exacerbated pulmonary inflammation.

### eG+CpG ODN does not induce Th2 responses in the lungs following RSV challenge

**Figure 5:**
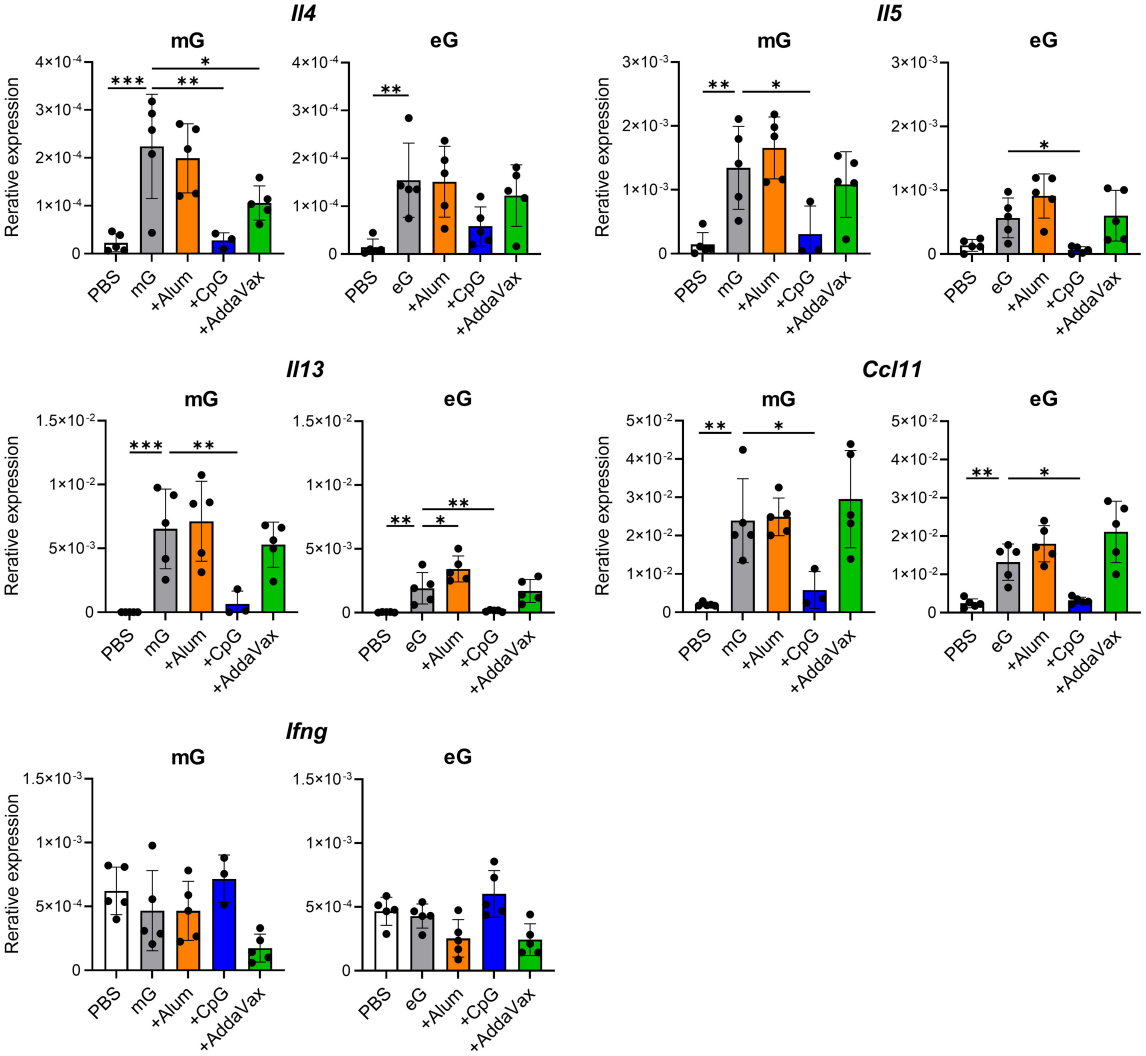
Gene expression in the lungs after RSV challenge following mG or eG vaccines. Mice immunized with mG or eG vaccines were challenged intranasally with 1.0 × 10^5^ PFU of RSV 10 days after the second immunization. At 5 days post-infection, the right lungs were excised, and the relative expression levels of *Il4, Il5*, *Il13, Ccl11*, and *Ifng* mRNA normalized to *Gapdh* were using real-time RT-PCR. This experiment was performed twice. n = 3–5 per group. Data are presented as the mean ± SD. ^*^ *P* < 0.05, ^**^ *P* < 0.01, ^***^ *P* < 0.001 as indicated using Dunnett’s test compared with mG alone or eG alone.

A characteristic feature of VAED in mouse models is cytokine expression, which is strongly skewed towards Th2 responses. Therefore, using real-time RT-PCR, we compared the mRNA expression of Th1 cytokine (i.e., *Ifng*) or Th2 cytokines (i.e., *Il4, Il5*, and *Il13*) and the chemokine *Ccl11* (eotaxin-1), which induces eosinophil migration (36, 37), in the lungs of vaccinated mice after RSV challenge (**Figure 5**). Compared with the PBS-treated group, mG and eG alone significantly increased the mRNA expression of Th2 cytokines and *Ccl11* but did not affect the mRNA expression of Th1 cytokines. The mRNA expression levels of Th2 cytokines and *Ccl11* were higher in the mG group than those in the eG group. However, the addition of CpG ODN significantly decreased the mRNA expression of Th2 cytokines and *Ccl11* compared to the antigen alone in either the mG or eG vaccines, while it did not increase the mRNA expression of Th1 cytokines. The addition of alum did not increase the mRNA expression of Th1 and Th2 cytokines or *Ccl11* in the mG vaccine but significantly increased the mRNA expression of *Il5* only in the eG vaccine. AddaVax did not increase the mRNA expression of Th1 and Th2 cytokines or *Ccl11* in either the mG or eG vaccines. These results correlated with the cytokine production profile of CD4^+^ T cells induced by the mG and eG vaccines (**Figure 2**). No notable differences in the mRNA expression of inflammatory cytokines (i.e., *Il6, Tnfa*, and *Il17a*) were observed in the lungs of vaccinated mice (**Supplementary Figure 5**). Thus, eG vaccines did not induce more Th2 responses than those by mG vaccines in the lungs after RSV challenge, and mG or eG+CpG ODN did not induce Th2 responses, whereas the other vaccines considerably did.

### The mixed eG and F vaccine with CpG ODN adjuvant provided enhanced protection against RSV challenge in the upper respiratory tract

**Figure 6:**
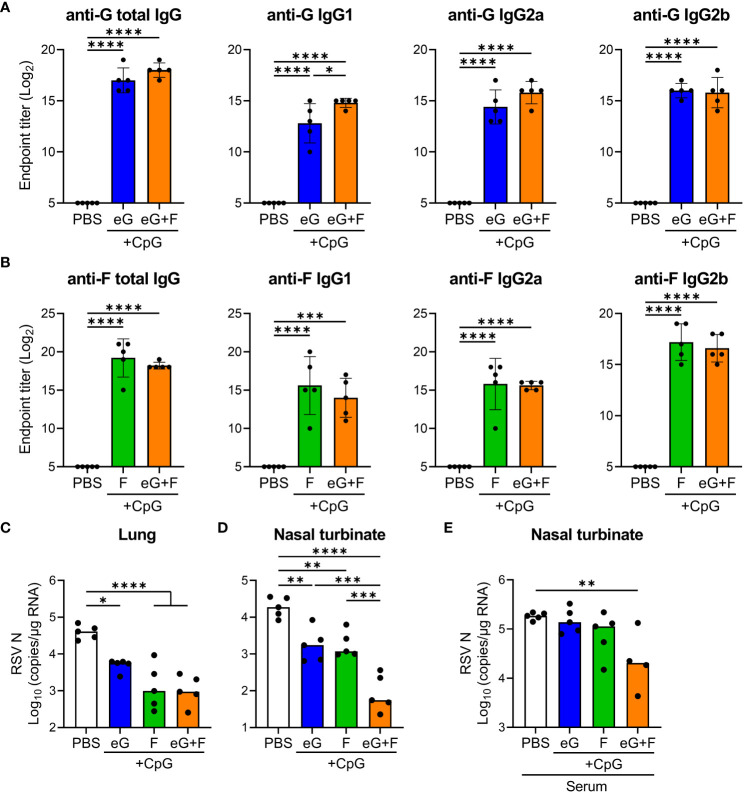
The protective effect of eG+F vaccine against RSV challenge. **(A, B)** The endpoint titers of **(A)** mG- or **(B)** F-specific total IgG, IgG1, IgG2a, and IgG2b in plasma were evaluated using ELISA, 7 days after second immunization with eG, F protein, or eG+F+CpG ODN. **(C, D)** Mice were challenged intranasally with 1.0 × 10^5^ PFU of RSV 10 days after the second immunization. At 5 days post-infection, **(C)** the lungs and **(D)** nasal turbinates were collected, and viral loads were determined using real-time RT-PCR for the mRNA levels of RSV nucleoprotein. **(E)** Mixture of 1.0 × 10^5^ PFU RSV and 4-fold diluted serum from PBS-treated control mice or eG- or F-immunized mice was administered intranasally into naive mice. At 5 days post-infection, the right lungs were excised, and viral loads were determined using real-time RT-PCR for the mRNA levels of RSV nucleoprotein. **(A–E)** Each experiment was performed **(A-D)** three times or **(E)** twice. **(A-D)** n = 5 per group. **(E)** n = 4–5 per group. Data are presented as **(A, B)** the mean ± SD or **(C-E)** the median. **(A-E)** ^*^ *P* < 0.05, ^**^ *P* < 0.01, ^***^ *P* < 0.001, ^****^ *P* < 0.0001 as indicated using Tukey’s test.

Finally, we evaluated the combined effects of the eG and F proteins (eG+F). To achieve this, we produced the F protein based on DS-Cav1 using mammalian cells. DS-Cav1 has a pre-F structure stabilized by amino acid mutations and can induce high levels of neutralizing antibodies (11). The F protein has a disulfide-linked structure of a F1 subunit containing a C-terminal foldon, His-tag (approximately 50 kDa), and F2 subunit (approximately 10 kDa) (11). SDS-PAGE identified an F protein that includes F1 and F2 subunits at approximately 60 kDa under non-reducing conditions and F1 at approximately 50 kDa under reducing conditions (**Supplementary Figure 1B**). Mice were vaccinated with eG+CpG ODN, F+CpG ODN, or eG+F+CpG ODN on days 0 and 21, and serum levels of G- and F-specific total IgG, IgG1, IgG2a, and IgG2b were measured on day 28 (**Figures 6A, B**). We confirmed that eG+CpG ODN induced significantly higher levels of G-specific total IgG, IgG1, IgG2a, and IgG2b than those in the PBS-treated group (**Figure 6A**), while F+CpG ODN induced significantly higher levels of F-specific total IgG, IgG1, IgG2a, and IgG2b levels than those in the PBS-treated group (**Figure 6B**). eG+F+CpG ODN induced significantly higher levels of G- and F-specific total IgG, IgG1, IgG2a, and IgG2b levels than those in the PBS-treated group (**Figures 6A, B**). In addition, compared to eG+CpG ODN, eG+F+CpG ODN significantly induced G-specific IgG1 (**Figure 6A**). Further, the protective effects of these vaccines against RSV challenge were compared. Compared with the PBS-treated group, eG+CpG ODN, F+CpG ODN, or eG+F+CpG ODN reduced the viral load in the lungs and nasal turbinates (**Figures 6C, D**). Although eG+CpG ODN did not reduce the viral load in the lungs compared to F+CpG ODN, it did reduce the viral load in the nasal turbinates to the same extent as F+CpG ODN (**Figures 6C, D**). Compared to eG+CpG ODN and F+CpG ODN, eG+F+CpG ODN significantly reduced the viral load in the nasal turbinates, although there was no difference in the lungs viral load between F+CpG ODN and eG+F+CpG ODN (**Figures 6C, D**). In addition, none of these vaccines increased the number of infiltrating cells in the lungs after the RSV challenge (**Supplementary Figure 6**). Thus, eG+F+CpG ODN significantly protected against RSV challenge in nasal turbinates relative to single antigen vaccines. Subsequently, to determine whether the combined effect of eG+F in the nasal turbinates was due to induced antibodies, serum from mice immunized with PBS, eG+CpG ODN, or F+CpG ODN was mixed with RSV and administered intranasally to naive mice (**Figure 6E**). Although serum from mice immunized with eG+CpG ODN or F+CpG ODN alone did not reduce the viral load in the nasal turbinates relative to that in PBS-treated mice, the serum mixture from mice immunized with eG+CpG ODN and F+CpG ODN significantly did (**Figure 6E**). This suggests that in the eG+F vaccine, both G- and F-specific antibodies protect against RSV challenge in the nasal turbinate. Thus, eG+CpG ODN could protect against RSV challenge comparable to F+CpG ODN in the nasal turbinates, and eG+F+CpG ODN could protect against RSV challenge more effectively than that by eG+CpG ODN or F+CpG ODN in the nasal turbinates.

## Discussion

This study demonstrated that non-glycosylated eG vaccines with or without an adjuvant, induced higher levels of antibodies and better attenuated the Th2 responses and eosinophilia after RSV challenge compared with the glycosylated mG vaccine (results are summarized in **Table 1**). In particular, the combination of eG and CpG ODN strongly induced G-specific antibodies and Th1 cells and protected against RSV challenge without inducing lung inflammation (**Table 1**). Moreover, compared to the respective single-antigen vaccines, eG+F+CpG ODN further protected against upper respiratory tract RSV challenge. Thus, our results indicate the efficacy and safety of eG for protection against RSV challenge. For enhanced safety, eG should be used in combination with Th1-type adjuvants such as CpG ODN, but not with other adjuvants such as alum or AddaVax.

Table 1Efficacy and adverse reactions in mG or eG vaccines.

**Table:** 

mG vaccine		mG alone	+Alum	+CpG	+AddaVax
Antibody*^1^		–	–	–	+
T cell*^2^	**Th1**	–	–	++	–
	**Th2**	++	+	–	+
Protection*^3^		+	+	–	++
Lung inflammation*^4^		++	++	–	++

**Table:** 

eG vaccine		eG alone	+Alum	+CpG	+AddaVax
Antibody*^1^		+	++	++	++
T cell*^2^	**Th1**	–	–	++	–
	**Th2**	+	+	–	–
Protection*^3^		+	++	++	++
Lung inflammation*^4^		–	–	–	+

*^1^Relative antibody levels were scored on a three-point scale based on the G-specific total IgG plasma levels: -: not detected, +: moderate levels, ++: high levels. *^2^The relative degree of T cell responses was scored on a three-point scale based on the percentage of CD4^+^ T cells producing Th1 cytokine (IFN-γ) or Th2 cytokines (IL-4, IL-5, and IL-13): -: not induced, +: moderately induced, ++: highly induced. *^3^Relative protection against RSV challenge was scored on a three-point scale based on lung viral load: -: little or no protection, +: moderate protection, ++: superior protection. *^4^Relative lung inflammation was scored on a three-point scale based on lung weight and infiltrating cell: -: little or no inflammation, +: moderate inflammation, ++: severe inflammation.

Our results are consistent with those of previous reports, which have compared the efficacy of mG and eG vaccines using Emulsigen, an oil-in-water nanoemulsion, as an adjuvant (17, 26). mG is modified by large amounts of O-linked glycans (38). These findings suggest that glycans in mG may induce Th2 cells and impede the induction of antibodies. Soluble glycosylated G proteins inhibit the activation of human dendritic cells by binding to the glycan recognition receptors DC-SIGN and L-SIGN *in vitro* (39). The binding of ligands to DC-SIGN has also contributes to the formation of a Th2-biased environment (40–42). In addition, the structural differences between mG and eG, including glycosylation as well as other post-translational modifications and folding, may contribute to immune responses. As G proteins are also known to bind CX3CR1 and heparan sulfate (2), the structural differences between mG and eG may affect their binding ability to CX3CR1and heparan sulfate, which may alter pharmacokinetics of mG or eG and cell migration, thereby affecting antibody production and T cell responses. Therefore, a more detailed analysis is required to clarify whether glycans on G proteins and the structures of mG and eG are involved in immunosuppression and T cell differentiation.

In this study, RSV challenge following serum transfer was suppressed in correlation with the levels of G-specific antibodies induced by the mG and eG vaccines. However, we were unable to identify whether the protection against RSV challenge was due to neutralizing or non-neutralizing antibodies. Antibodies against CCD containing the CX3C motif possess neutralizing activity *in vitro* (19–21, 23). In contrast, antibodies against non-CCD contribute to viral elimination in a non-neutralizing manner, including antibody-dependent cellular cytotoxicity (ADCC) and antibody-dependent cellular phagocytosis, although they lack neutralizing activity (43). The G protein produced in *E. coli* has been shown to induce higher levels of antibodies against CCD and non-CCD than those by the G protein produced in mammalian cells (17). Thus, the eG vaccine protects against RSV challenge by inducing both neutralizing and non-neutralizing antibodies. In addition, as previously reported that the addition of CpG ODN induces antigen-specific IgG2 in mice (25, 28), eG+CpG ODN strongly induced G-specific IgG2. Because IgG2 has high ADCC activity in mice (44, 45), eG+CpG ODN may provide enhanced protection against RSV challenge with non-neutralizing antibodies. Thus, eG+CpG ODN vaccines are considered promising candidates for inducing antibodies that protect against RSV.

mG vaccines, except mG+CpG ODN, protected against RSV challenge in the lungs, despite the low antibody titer. In contrast, a decrease in viral load was not observed on administering serum from mice vaccinated with mG+alum, compared to those vaccinated with eG+alum or CpG ODN. Therefore, factors other than antibodies may be involved in protecting against RSV challenge. One such factor is eosinophils. mG+alum or Addavax induced eosinophils in the lungs after the RSV challenge. Because eosinophils express TLR7, which recognizes single-stranded RNA, eosinophils recognize RSV via TLR7 and produce type I IFN and nitric oxide to promote antiviral responses in mice (46, 47). Therefore, eosinophils migrating to the lungs after RSV challenge following mG vaccination may promote pulmonary inflammation while eliminating the virus.

RSV-specific CD8^+^ T cells protect against RSV infection (48). The addition of CpG ODN to vaccines induces antigen-specific CD8^+^ T cells (49). Our results also showed that mG or eG+CpG ODN induced a similar level of G-specific IFN-γ-producing CD8^+^ T cells. However, eG+CpG ODN protected against RSV challenge in the lungs, but mG+CpG ODN did not. Therefore, G-specific CD8^+^ T cells may not contribute to the protection against RSV challenge in our model.

We have demonstrated that eG+F+CpG ODN is more effective than eG or F+CpG ODN in protecting against RSV challenge in the upper respiratory tract. Other studies have reported that the combination vaccine of recombinant G and F proteins protects against lower respiratory tract RSV challenge comparable to each single antigen vaccine, whereas protection against upper respiratory tract RSV challenge has not been evaluated (21, 25). Therefore, F protein vaccines can be further improved by enhancing its efficacy in preventing RSV challenge in the upper respiratory tract, which is the primary site of RSV infection (50). The efficacy of F protein vaccines against RSV-related acute respiratory infections is low (14, 15). Therefore, the eG+F vaccine would complement the insufficiency of F protein vaccines in protecting against upper respiratory tract infections, thus preventing the spread of RSV and controlling community transmission. However, it is unclear why the eG+F vaccine provides better protecting against RSV challenge than that by single-antigen vaccine alone in the upper respiratory tract. The present study findings suggest that the response of G and F proteins to RSV infection may vary in the upper and lower respiratory tracts, and the distribution of RSV infection receptors, such as CX3CR1, TLR4, and nucleolin, may show varied expression. Therefore, G protein vaccines will primarily focus on preventing upper respiratory tract infections, that is, the infection itself and its transmission.

In conclusion, we found that eG+CpG ODN induced G-specific IgG and Th1 cells, and protected against RSV challenge without pulmonary inflammation. Thus, the use of Th1-type adjuvants, such as CpG ODN, are an effective strategy to avoid adverse reactions to G protein vaccines. However, our study has some limitations. Using size exclusion gel filtration chromatography and SDS-PAGE we confirmed mG and eG purity, along with low endotoxin levels. However, because each protein may contain mammalian cell- or *E. coli-*derived compound trace amounts, the effects of these components on vaccine efficacy should be investigated in the future. Furthermore, the CpG ODN-induced immune response in mice may be overestimated because TLR9-expressing cells are restricted in humans (51). Therefore, vaccine efficacy with the addition of other Th1-type adjuvants, such as monophosphoryl lipid A, should be explored. In addition, because we used naive adult mice, the results of this study cannot be directly extrapolated to pregnant women, older adults with repeated RSV infections, or to infants, and should be confirmed in clinical trials. We believe that our results provide valuable information for the clinical applicability of G protein vaccines.

## Data availability statement

The original contributions presented in the study are included in the article/**Supplementary Material**. Further inquiries can be directed to the corresponding author.

## Ethics statement

The animal study was approved by the guidelines of Osaka University for the ethical treatment of animals and were approved by the Animal Care and Use Committee of the Research Institute for Microbial Diseases, Osaka University, Japan (protocol numbers: BIKEN-AP-R01-15-2, BIKEN-AP-R02-14-5). The study was conducted in accordance with the local legislation and institutional requirements.

## Author contributions

EK: Formal Analysis, Funding acquisition, Investigation, Writing – original draft, Writing – review & editing. TS: Investigation, Methodology, Resources, Writing – review & editing. TH: Investigation, Writing – review & editing. YY: Conceptualization, Funding acquisition, Project administration, Supervision, Writing – original draft, Writing – review & editing.
