# Supplementary figures and images for: Non-glycosylated G protein with CpG ODN provides robust protection against respiratory syncytial virus without inducing eosinophilia
Source: Front Immunol. 2023 Dec 19;14:1282016. doi: 10.3389/fimmu.2023.1282016 (PMC10758452; doi:10.3389/fimmu.2023.1282016)

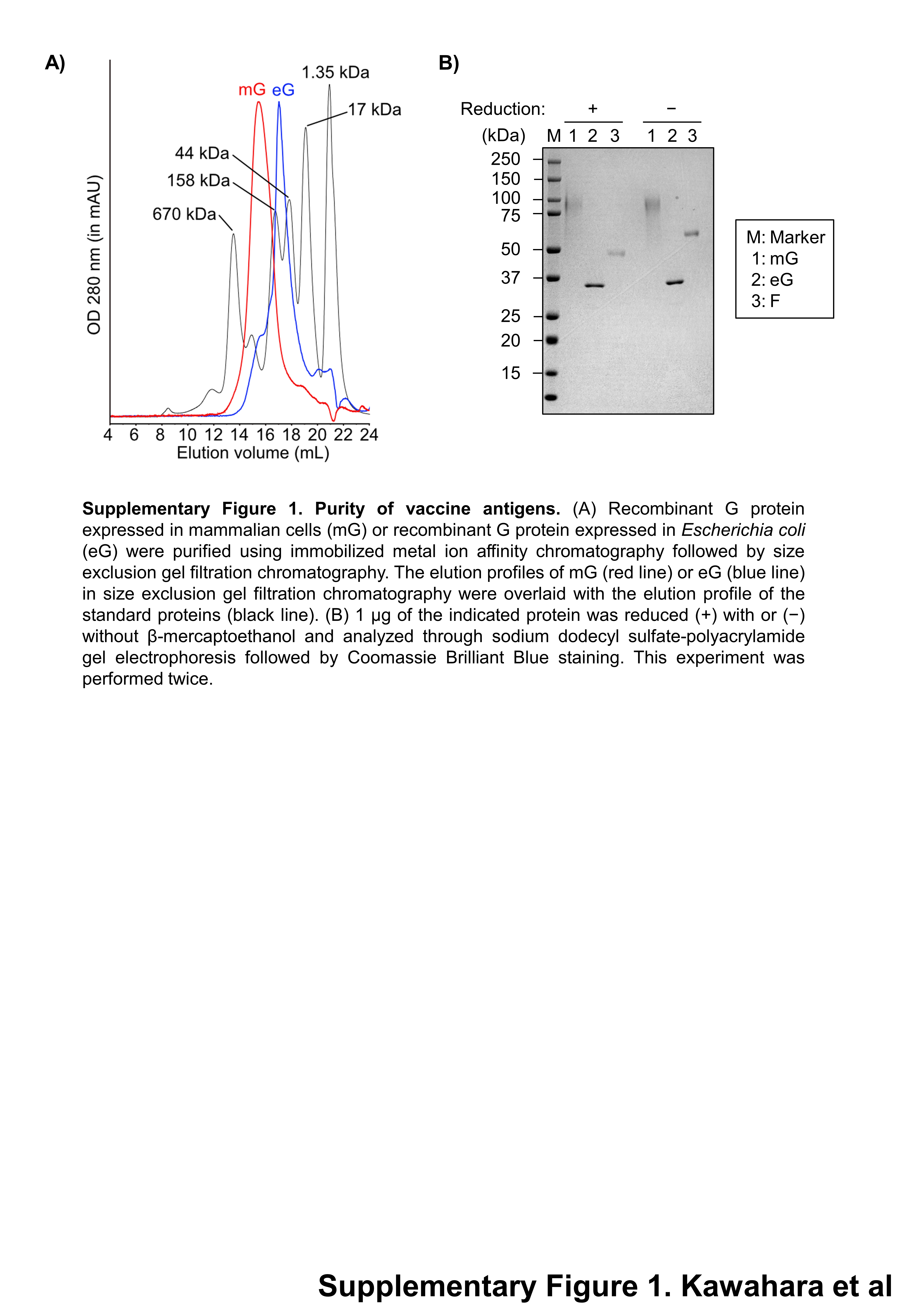

Supplement: Supplementary file 1 [file Image_1.tif]

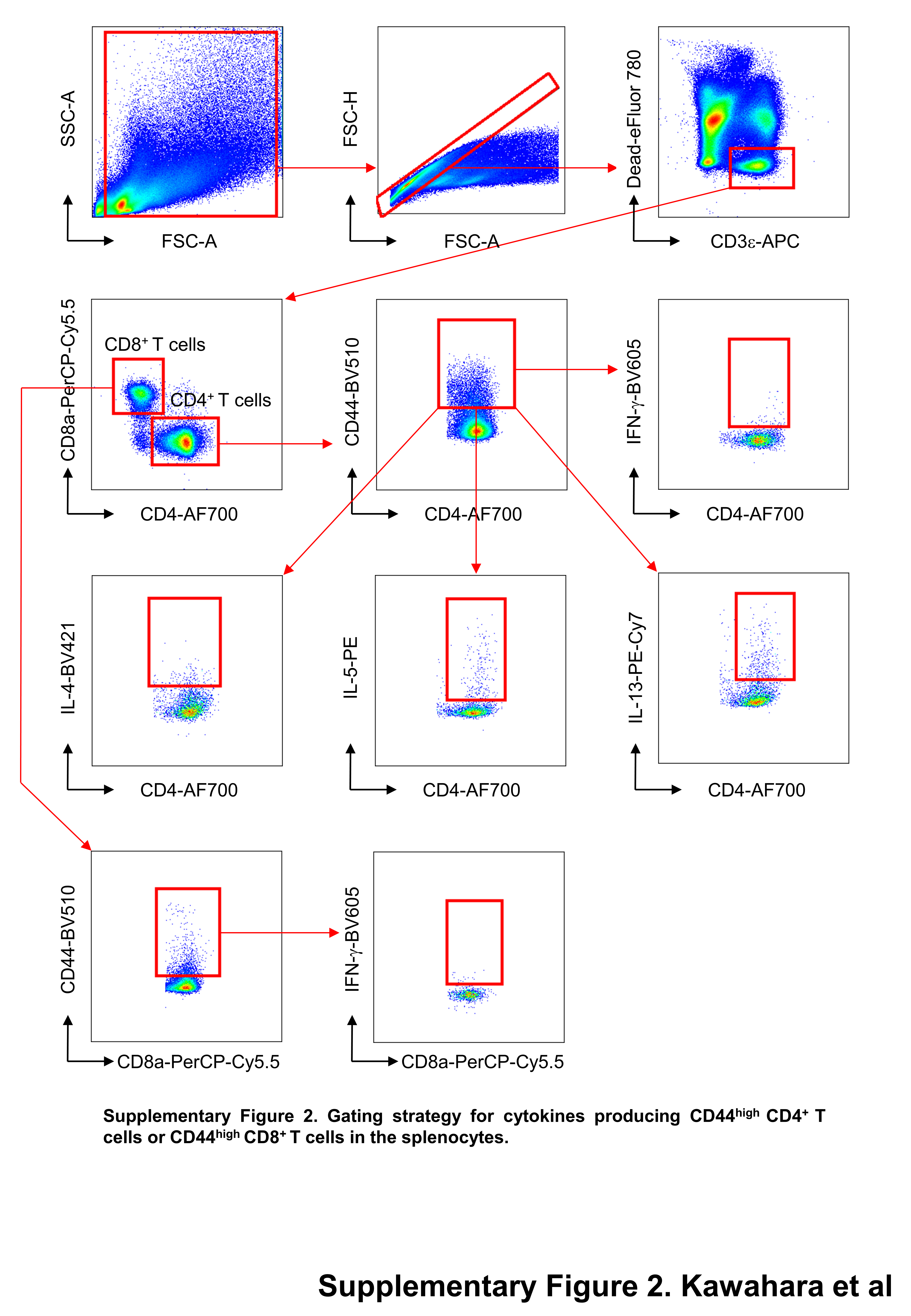

Supplement: Supplementary file 2 [file Image_2.tif]

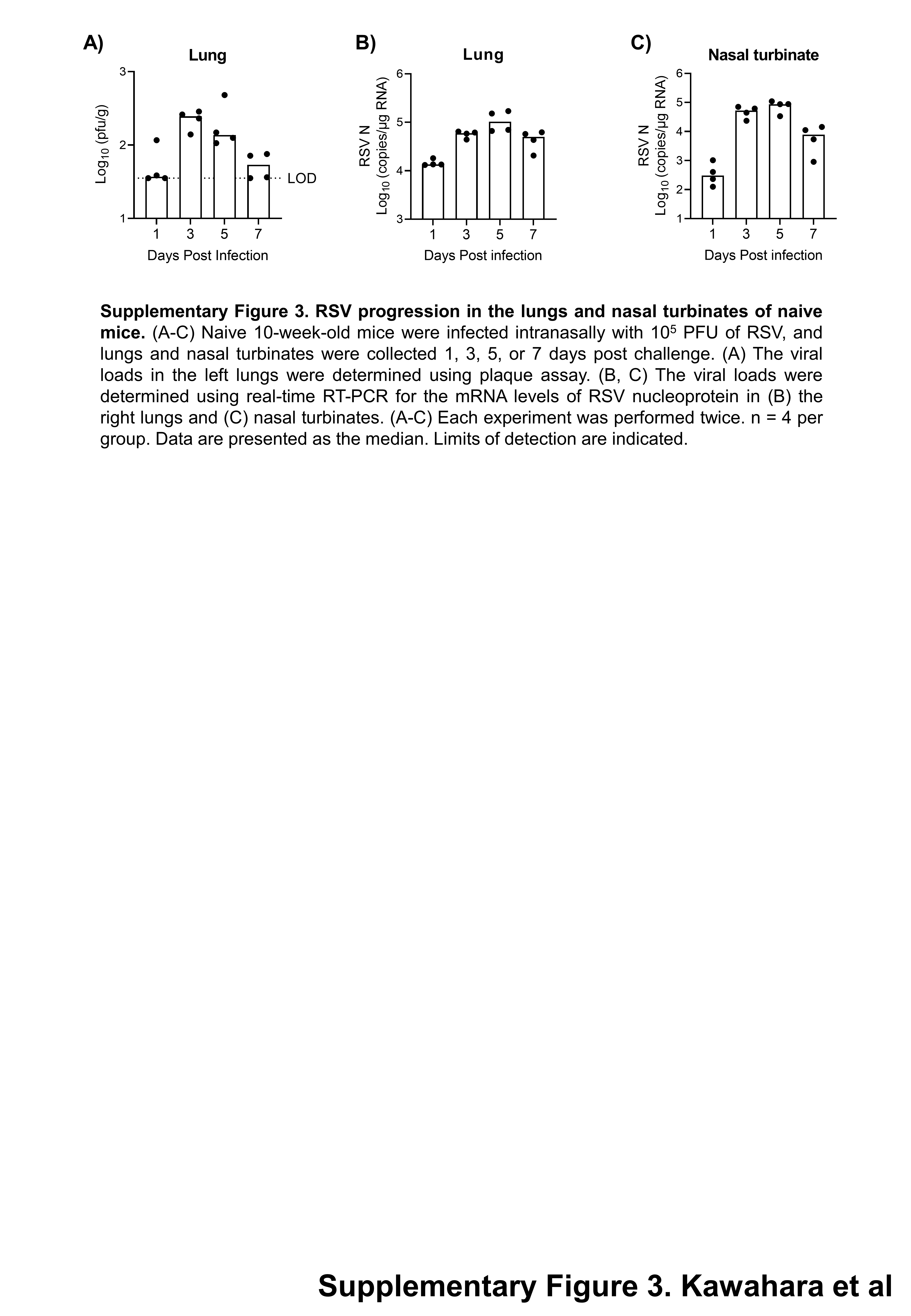

Supplement: Supplementary file 3 [file Image_3.tif]

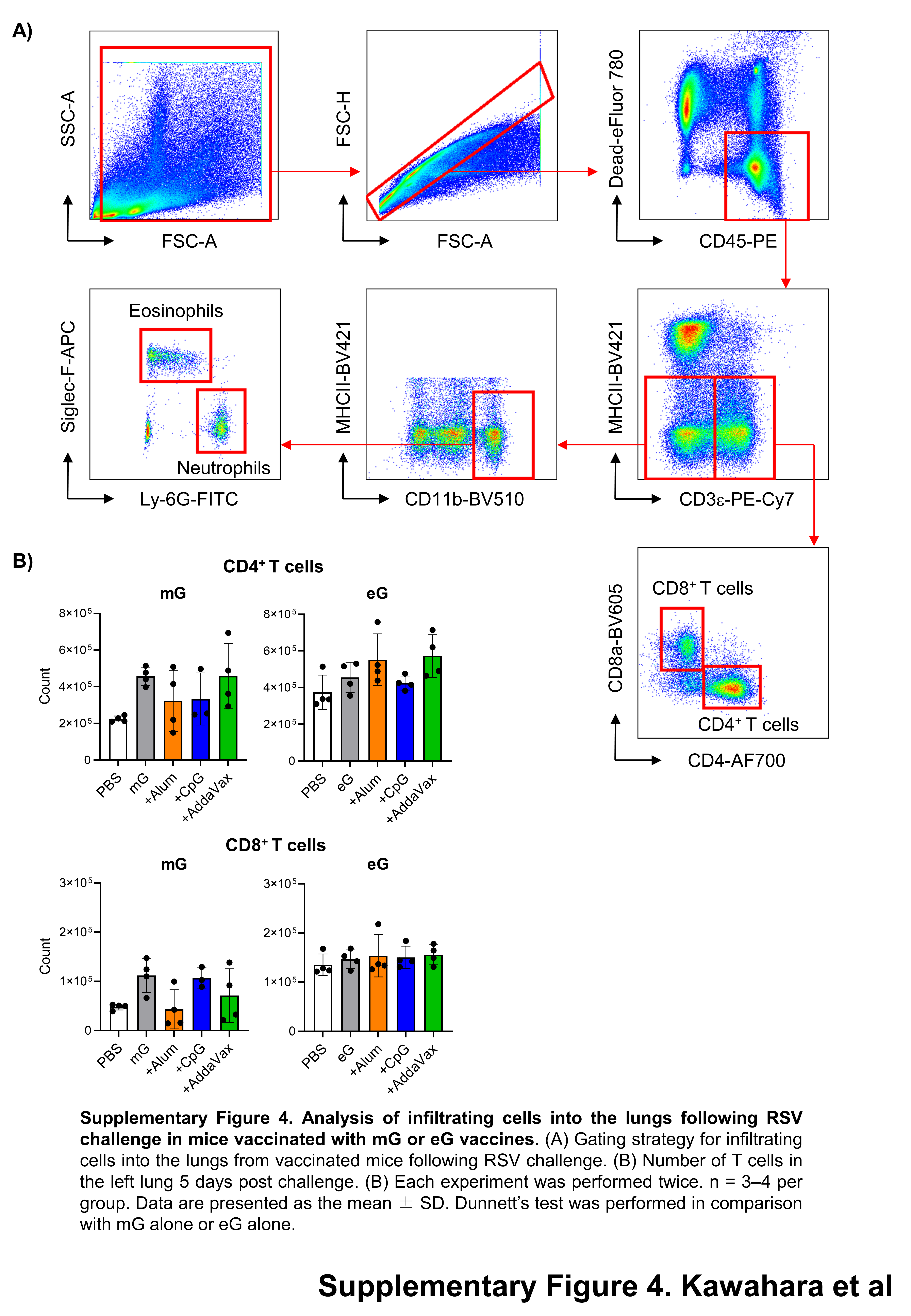

Supplement: Supplementary file 4 [file Image_4.tif]

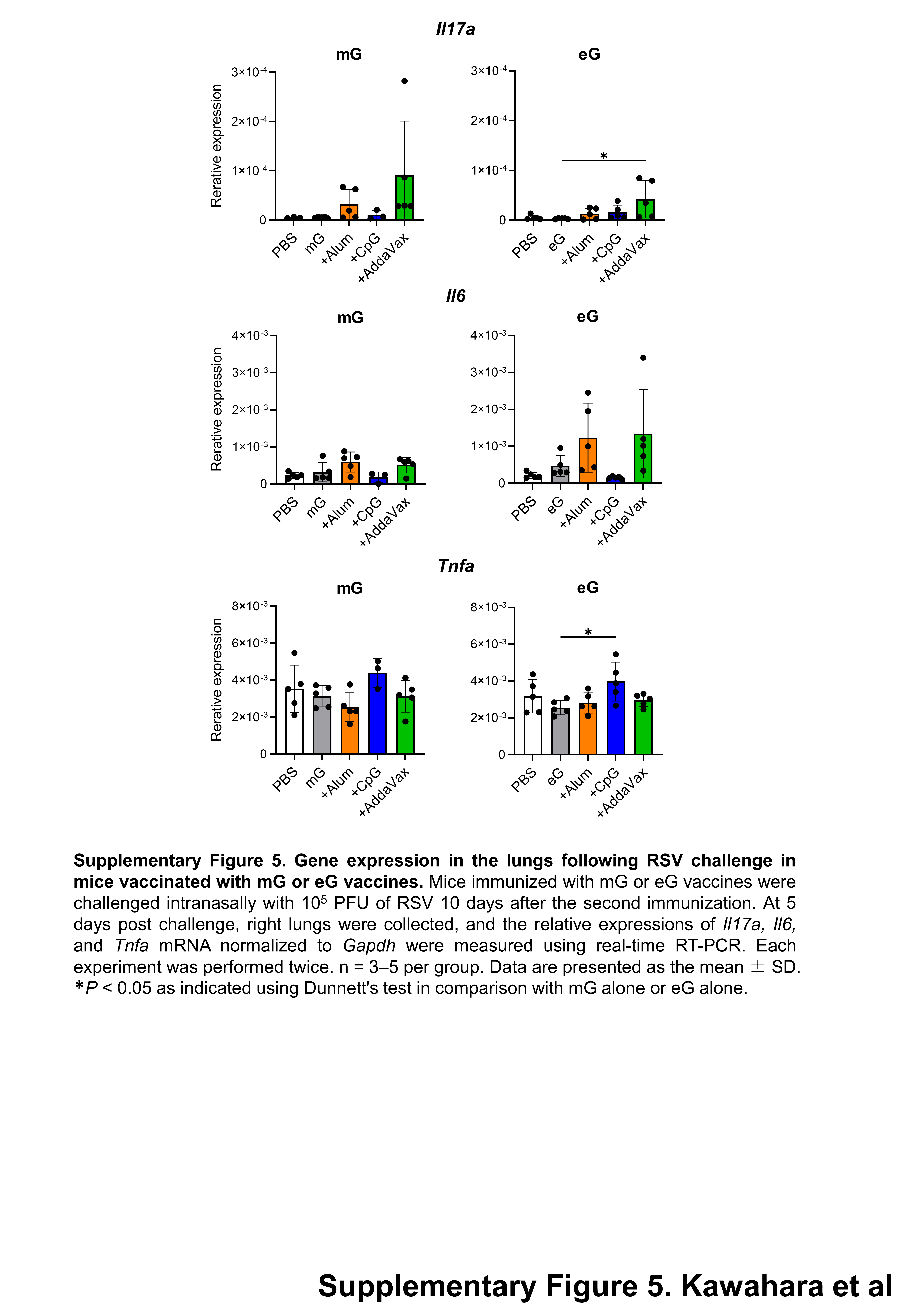

Supplement: Supplementary file 5 [file Image_5.tif]

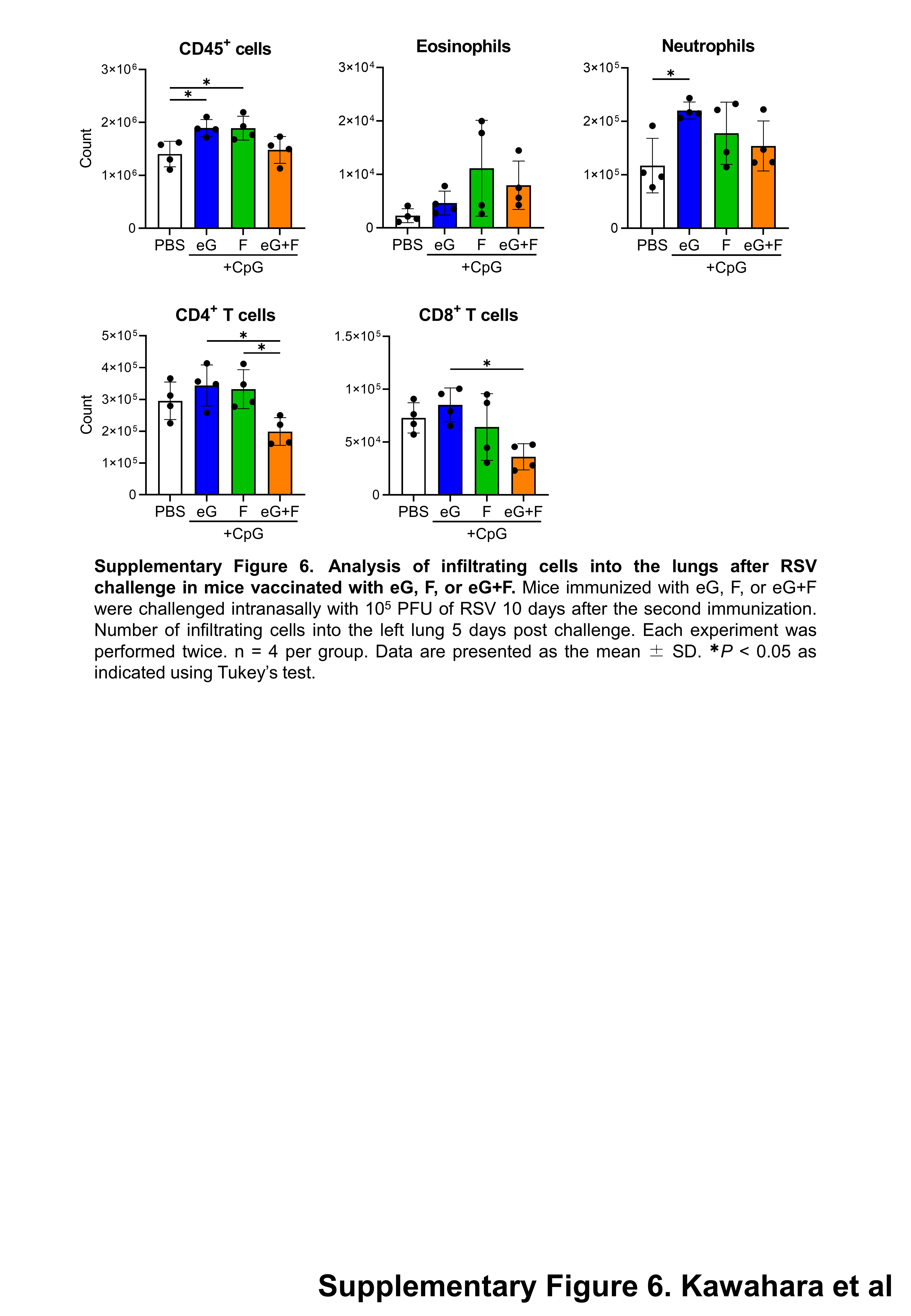

Supplement: Supplementary file 6 [file Image_6.tif]

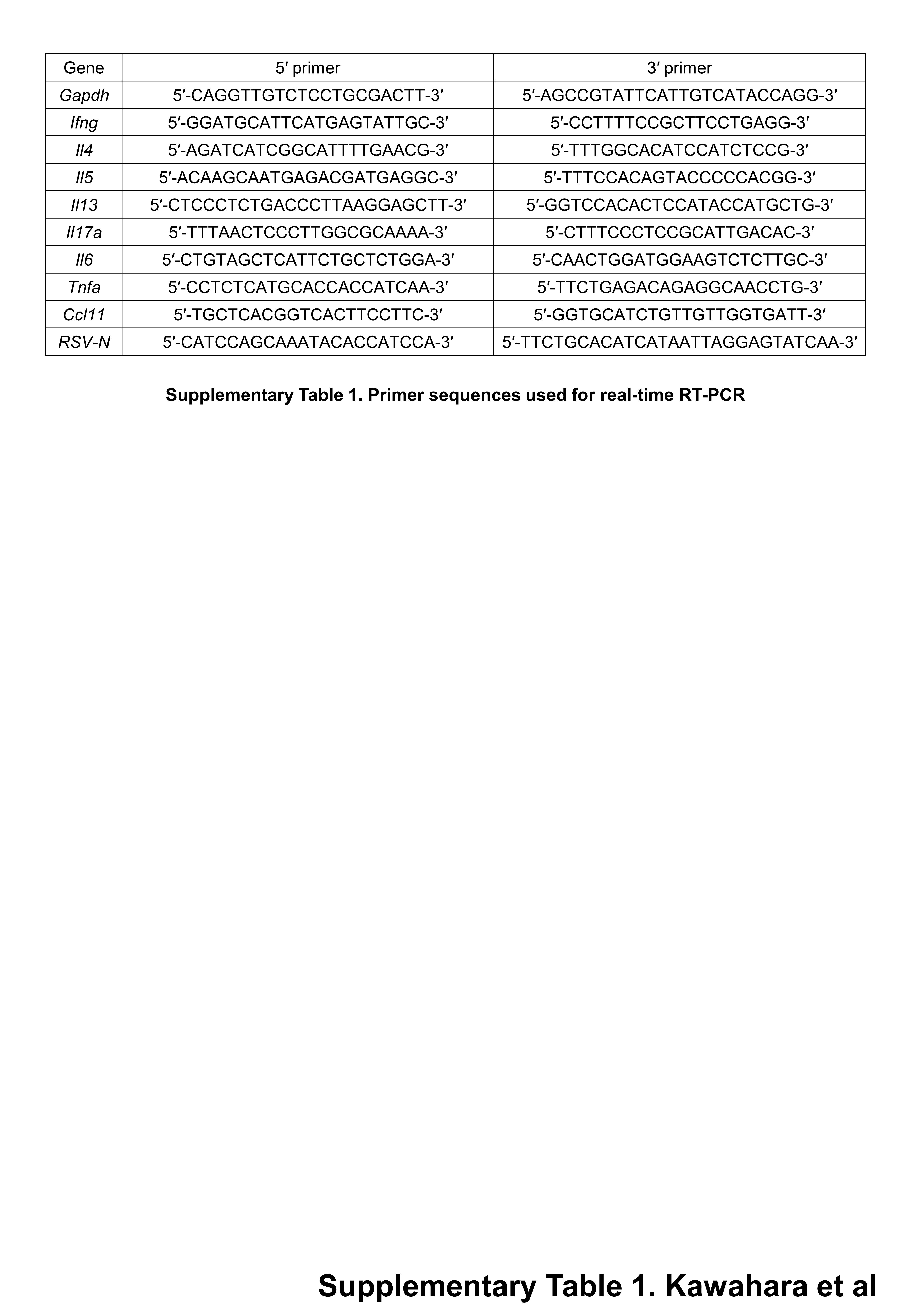

Supplement: Supplementary file 7 [file Image_7.tif]
